# Ultraviolet-B enhances the resistance of multiple plant species to lepidopteran insect herbivory through the jasmonic acid pathway

**DOI:** 10.1038/s41598-017-18600-7

**Published:** 2018-01-10

**Authors:** Jinfeng Qi, Mou Zhang, Chengkai Lu, Christian Hettenhausen, Qing Tan, Guoyan Cao, Xudong Zhu, Guoxing Wu, Jianqiang Wu

**Affiliations:** 1 Department of Economic Plants and Biotechnology, Yunnan Key Laboratory for Wild Plant Resources, Kunming Institute of Botany, Chinese Academy of Sciences, Kunming, 650201 China; 2 College of Plant Protection, Yunnan Agriculture University, Kunming, 650201 China; 3 State Key Laboratory of Rice Biology, China National Rice Research Institute, Hangzhou, 31006 China

## Abstract

Land plants protect themselves from ultraviolet-B (UV-B) by accumulating UV-absorbing metabolites, which may also function as anti-insect toxins. Previous studies have shown that UV-B enhances the resistance of different plant species to pierce-sucking pests; however, whether and how UV-B influences plant defense against chewing caterpillars are not well understood. Here we show that UV-B treatment increased *Spodoptera litura* herbivory-induced jasmonic acid (JA) production in Arabidopsis and thereby Arabidopsis exhibited elevated resistance to *S*. *litura*. Using mutants impaired in the biosynthesis of JA and the defensive metabolites glucosinolates (GSs), we show that the UV-B-induced resistance to *S*. *litura* is dependent on the JA-regulated GSs and an unidentified anti-insect metabolite(s). Similarly, UV-B treatment also enhanced the levels of JA-isoleucine conjugate and defense-related secondary metabolites in tobacco, rice, and maize after these plants were treated with simulated herbivory of lepidopteran insects; consistently, these plants showed elevated resistance to insect larvae. Using transgenic plants impaired in JA biosynthesis or signaling, we further demonstrate that the UV-B-enhanced defense responses also require the JA pathway in tobacco and rice. Our findings reveal a likely conserved JA-dependent mechanism by which UV-B enhances plant defense against lepidopteran insects.

## Introduction

Insect feeding is one of the major biotic factors threatening plant survival, and during coevolution, plant have developed complex defense systems to contend with insect herbivores^1^. After sensing wounding inflicted by insect feeding or perceiving certain elicitors in the oral secretions of insects, plants activate signaling networks, such as Ca^2+^, mitogen-activated protein kinase (MAPK), and phytohormone signaling cascades^1–3^, to modulate the biosynthesis of defense-related metabolites. Downstream of all of these signaling cascades, JA signaling specifically regulates plant responses to wounding and herbivory^4^. Both monocotyledons and dicotyledons impaired in JA biosynthesis or perception are highly susceptible to lepidopteran caterpillars^5,6^. Importantly, genetic and biochemical evidence revealed that JA-Ile (JA-isoleucine conjugate), instead of JA itself, induces many JA responses against herbivory^4,7^, and plants impaired in JA-Ile accumulation are very susceptible to lepidopteran insect feeding^8,9^.

It has been ~ 400 million years since plants colonized land to receive better light^10^. In the spectrum of sunlight that reaches the earth surface, ultraviolet-B (UV-B; 280 to 315 nm) has the highest energy which is detrimental to macromolecules, such as proteins and DNA^11^. One of the most important protective mechanisms in higher plants is to produce UV-absorbing compounds^3,12^, and these compounds may also function in plant interaction with other organisms, including pathogens and insects. In Arabidopsis, UV-B induced the accumulation of sinapate, which confers resistance to the necrotrophic fungus *Botrytis cinerea*, and this process was found to be controlled by UVR8, the receptor of UV-B^13,14^. Solar UV-B caused accumulation of 17-hydroxygeranyllinalool diterpene glycosides (HGL-DTGs) in the wild tobacco *Nicotiana attenuata*, and the increased HGL-DTGs deterred the sap-sucking bug *Tupiocoris notatus* ^15^. UV-B also enhanced the defense of broccoli plants against cabbage aphid *Brevicoryne brassicae*, probably by increasing flavonoid concentrations^16^. Some evidences have also shown that UV-B changes plant resistance to chewing insects. Under the field conditions, soybean plants were less damaged by chewing herbivores under ambient UV-B than under reduced UV-B condition^17^, and the weights of *Plutella xylostella* pupae were smaller on UV-radiated broccoli plants (*Brassica oleracea*)^18^. UV-B treatment positively affected the resistance of white clover (*Trifolium repens*) to *Spodoptera litura* ^19^. *Nicotiana attenuata* and *N*. *longiflora* exposed to UV-B were better defended against *Manduca sexta* than were the non-UV-B-treated plants, and this may be due to the UV-B-induced phenolic metabolites^20,21^.

Thus far, the underlying mechanisms by which UV-B affects plant resistance to insect herbivores are still not well understood^3,12,22^, although some evidences have suggested the implication of the JA pathway. UV-B-treated Arabidopsis showed increased levels of basal JA and *PDF1*.*2* transcripts^23^. In Arabidopsis, the UV-B-induced of phenolic compounds deterred the oviposition of *P*. *xylostella* and this effect was absent in the *jar1-1* (*jasmonate resistance-1*) mutant, which is deficient in JA-Ile^24^. Likewise, UV-B elevated levels of JA in *N*. *attenuata*, when induced by simulated *M*. *sexta* herbivory^15,20^. However, genetic evidence is still lacking to support the role of the JA pathway in UV-B-induced defense against chewing caterpillars^22^. Furthermore, it is unclear whether UV-B positive affects insect resistance in monocots, and if so, by what mechanisms.

Given the central role of JA signaling in plant resistance to chewing insect herbivores^4^, we hypothesized that UV-B may elevate plant resistance to chewing insects by affecting the JA signaling pathway. By comparing UV-B-treated and untreated plants, we show that UV-B radiation on Arabidopsis, tobacco (*Nicotiana tabacum*), rice (*Oryza sativa*), and maize (*Zea mays*) plants elevated these plants’ JA/JA-Ile levels, defensive secondary metabolites, and resistance to lepidopteran chewing insects. Using genetically modified plants silenced in JA biosynthesis or signaling, we further demonstrate that the JA pathway plays a major role in enhancing UV-B-pretreated plants’ defense against chewing caterpillars, and this mechanism is likely to be conserved in both mono- and dicots.

## Results

### JA pathway plays a major role in enhancing UV-B- pretreated Arabidopsis resistance to *S*. *litura*

**Figure 1:**
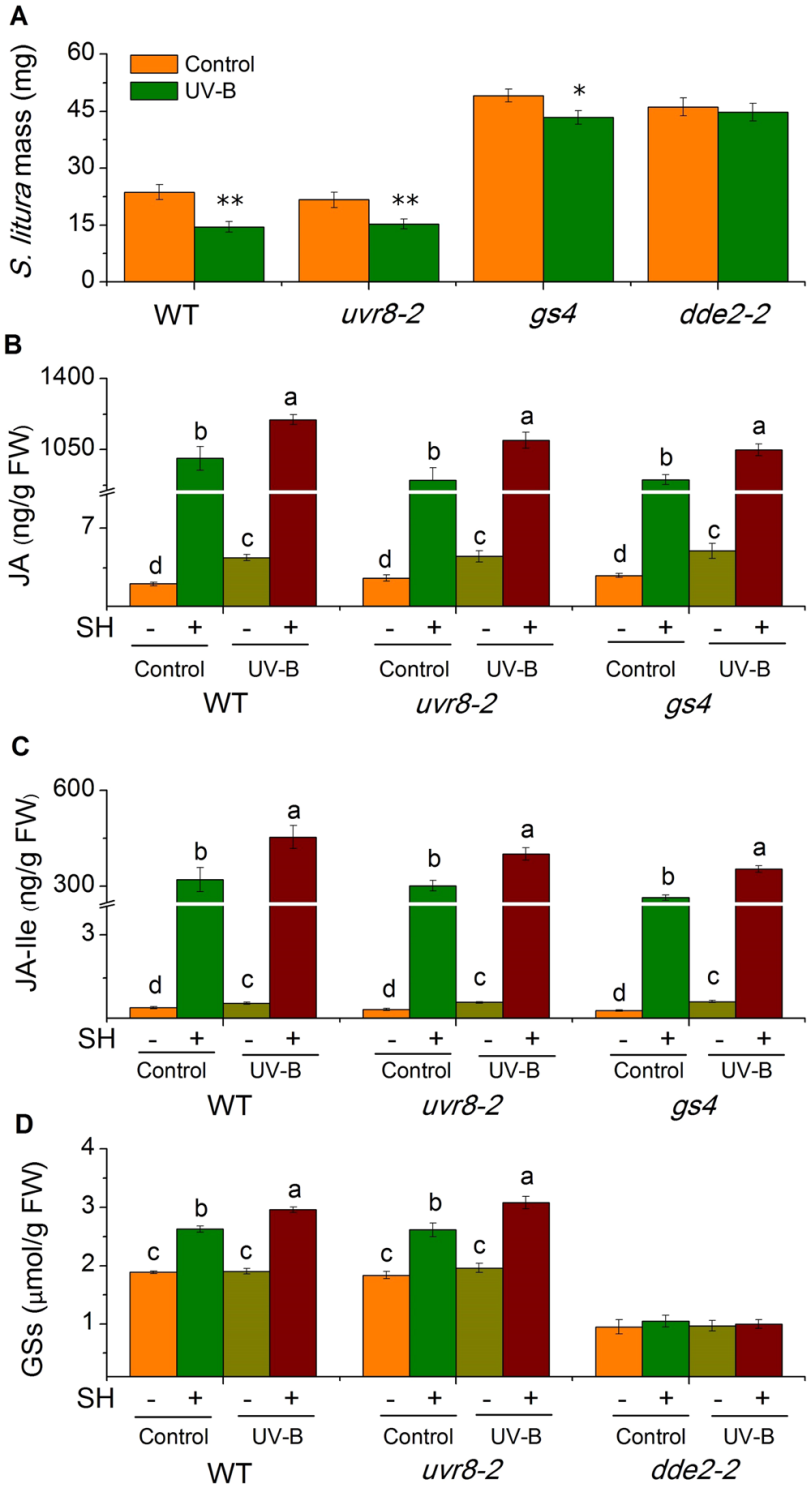
JA pathway is required for UV-B-enhanced Arabidopsis resistance to *S*. *litura*. (**A**) Mean masses (±SE) of *S*. *litura*, after 12 days of feeding on control and UV-B-pretreated wild-type (WT), *uvr8-2*, *gs4*, and *dde2-2* Arabidopsis. Control and UV-B-pre-exposed WT and mutant Arabidopsis were treated with simulated herbivory (SH+) or left untreated (SH−), and the levels of JA (**B**), JA-Ile (**C**), and total GSs (**D**) were determined in samples collected at 2 h (for B and C) or 48 h (for D). For A, n = 30-50; for B–D, n = 6–8. Asterisks (*P < 0.05, **P < 0.01, Student’s *t*-test) and different letters (*P* < 0.05, Duncan’s multiple range test) indicate significant differences between treatments within the same genotype.

To determine whether UV-B enhances Arabidopsis resistance to lepidopteran insects, we first treated Arabidopsis with UV-B for five days, and left the control group untreated under the same PAR (photosynthetically active radiation); thereafter, all plants were infested with *S*. *litura* neonates. After twelve days of feeding, *S*. *litura* reared on UV-B-treated plants weighed 39% less than those reared on the control group (Fig. 1A).

GSs are the major defensive compounds in Arabidopsis and are regulated by the JA signaling pathway^25^. To examine the pathways and metabolites that mediate UV-B-dependent Arabidopsis resistance to *S*. *litrua*, we repeated these experiments using *uvr8-2* (a UV-B receptor mutant), *gs4*, which is a quadruple mutant *cyp79B2 cyp79B3 myb28 myb29* impaired in GS biosynthesis (for simplicity, named *gs4*), and *dde2-2*, which has a mutation in the JA biosynthetic gene *AtAOS*. Similar to the WT (wild-type) plants, *uvr8-2* and *gs4* mutants also exhibited elevated resistance to *S*. *litura* in the UV-B-treated group (Fig. 1A), indicating that *UVR8* is not involved in UV-B-enhanced defense and that certain metabolites that were regulated by UV-B, but not GSs, conferred resistance to *S*. *litura*. Importantly, the effect of UV-B on Arabidopsis resistance to *S*. *litur*a diminished in the *dde2-2* mutant (Fig. 1A). Thus, the JA pathway plays an important role in UV-B-enhanced defense against *S*. *litura* in Arabidopsis.

To better understand the molecular and chemical mechanisms underlying the increased resistance to *S*. *litura* in UV-B pretreated Arabidopsis, we next determined the concentrations of phytohormones JA and JA-Ile, the defensive metabolites GSs, and the transcript levels of their biosynthesis-related genes. Given that insect feeding behavior is very hard to control, simulated herbivory (SH) was used to induce plants. Without SH, Arabidopsis plants in the UV-B-treated group had 30 and 19% higher levels of JA and JA-Ile, respectively, than did the plants in the control group. Two hours after SH treatment, the UV-B-treated group had even higher concentrations of JA and JA-Ile (33 and 42% increased, respectively) (Fig. 1B,C). Consistently, the basal and SH-induced levels of *AtOPR3* (JA biosynthesis-related) and *AtJAR1* (JA-Ile biosynthesis-related) were higher in the UV-B-treated group than in the control group (see Supplementary Fig. S1). When untreated with SH, UV-B pre-exposure did not affect GS accumulation in WT Arabidopsis plants, while after SH, the UV-B-treated group showed 13% higher levels of GSs than did the control group, indicating UV-B had a synergistic effect on herbivory-induced GS accumulation (Fig. 1D); in contract, in *dde2-2* mutant, GS levels were not induced by either UV-B pretreatment or SH (Fig. 1D).

These data indicate that UV-B pretreatment enhances the levels of herbivory-induced JA/JA-Ile in Arabidopsis plants, and thus elevated concentrations of GSs and another defensive metabolite(s), resulting in enhanced defense against *S*. *litura*. Thus, UV-B positively affects the resistance of Arabidopsis to *S*. *litura* through the JA pathway.

### UV-B treatment enhances tobacco defense against *S*. *litura* by elevating herbivory-induced JA and JA-Ile

**Figure 2:**
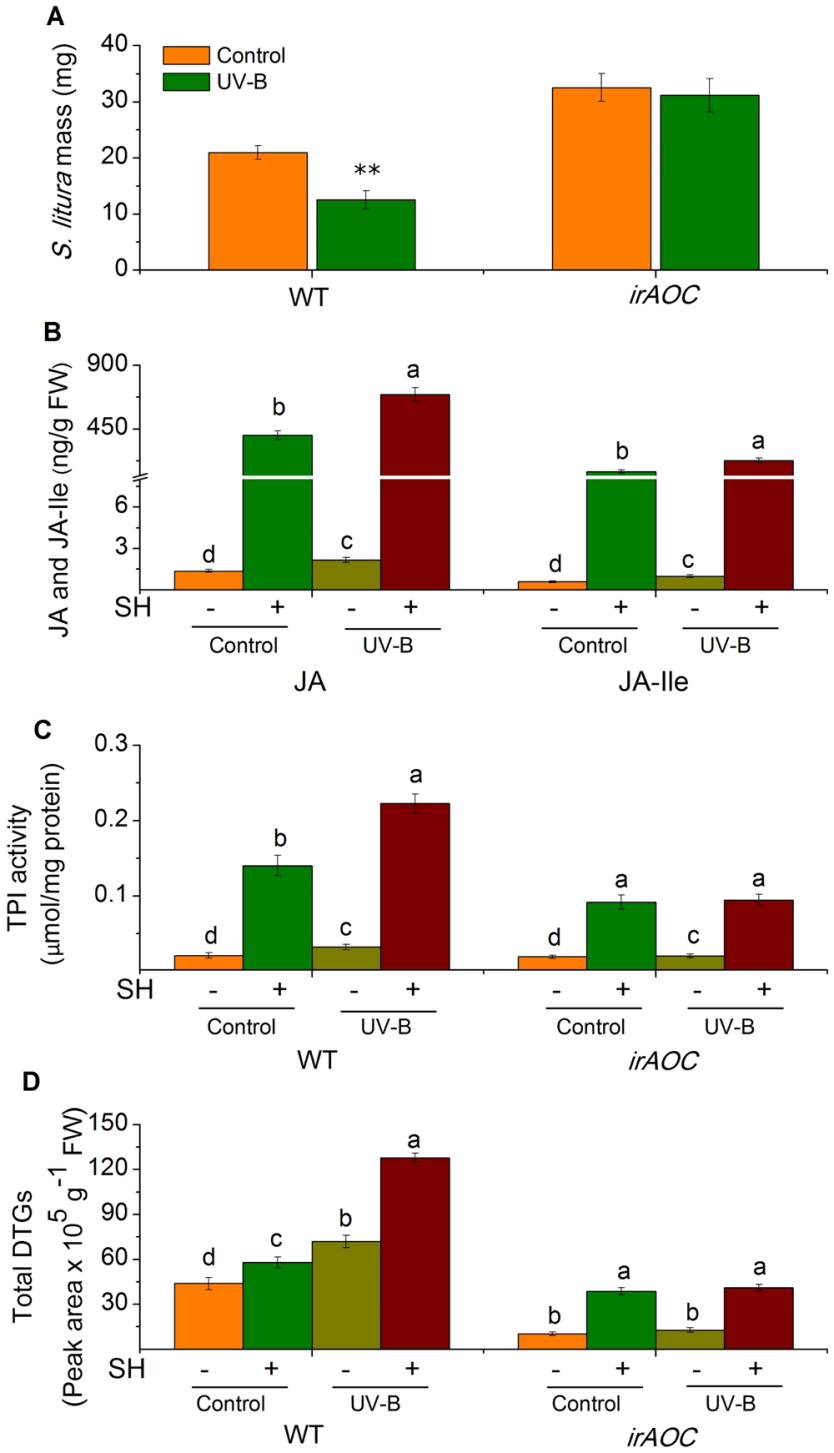
JA pathway is essential for UV-B-induced *N*. *tabacum* defense against *S*. *litura*. (**A**) Mean masses (±SE) of *S*. *litura*, eight days after feeding on control and UV-B-pretreated wild type (WT) and irAOC tobacco plants. Control and UV-B-pre-exposed WT and irAOC tobacco plants were treated with simulated herbivory (SH+) or left untreated (SH−), and the levels of JA and JA-Ile (**B**), TPI activity (**C**), and total DTGs (**D**) were quantified in samples harvested at 2 h (for B) or 48 h (for C and D). For A, n = 30–50; for B–D, n = 6–8. Asterisks (**P < 0.01, Student’s *t*-test) and different letters (*P* < 0.05, Duncan’s multiple range test) indicate significant differences between treatments within the same genotype (for A, C, D) or group of compounds (for B).

To examine whether UV-B increases plant resistance to lepidopteran caterpillars by affecting JA/JA-Ile in other plant species, *N*. *tabacum* plants were treated with 3 kJ m^−2^d^−1^ UV-B for five days followed by infestation of *S*. *litura*. Compared with those fed on control plants, *S*. *litura* insects gained 40% less weight on tobacco pretreated with UV-B (Fig. 2A), indicating a strong positive effect of UV-B. To further assess the function of the JA pathway in UV-B-regulated defense in tobacco, we measured the growth of *S*. *litura* fed on irAOC (deficient in JA) tobacco plants with or without UV-B-pretreatment. *S*. *litura* larvae gained the same weight regardless of whether irAOC was pretreated with UV-B or not (Fig. 2A). In WT tobacco plants, SH-induced JA and JA-Ile levels in the UV-B-pretreated group were 71 and 52% increased, respectively, compared with those in the control group (Fig. 2B). A similar pattern was also observed in the expression levels of *NtAOC* and *NtJAR* (see Supplementary Fig. S2A), which are a JA and a JA-Ile biosynthesis-related gene, respectively. These findings confirmed that UV-B enhances tobacco defense against *S*. *litura* and this is also regulated by the JA pathway, suggesting a conserved mechanism between Arabidopsis and tobacco.

We identified the major secondary metabolites in tobacco plants. The basal and SH-induced activity of trypsin protein inhibitors (TPIs) in the samples pretreated with UV-B was 54 and 59% higher than in control groups. Furthermore, increased basal and SH-induced HGL-DTGs were also found in the UV-B treatment group (Fig. 2C,D). Consistent with fact that *S*. *litura* growth was not affected by UV-B on irAOC tobacco plants, TPI activity and the contents of HGL-DTGs in irAOC tobacco were similarly induced by SH in the UV-B-pretreated and control group (Fig. 2C,D), indicating that the JA pathway is required for UV-B-boosted accumulation of TPIs and HGL-DTGs.

UV-B-pretreated tobacco plants showed increased levels of chlorogenic acid (CA), rutin, and dicaffeoylspermidine (DCS), while nicotine levels remained unaffected. (see Supplementary Fig. S2B–E). Although in irAOC plants, CA, rutin, and DCS were still higher in the UV-B-pretreated group than in the control group, the similar growth of *S*. *litura* on the UV-B-pretreated and control irAOC plants ruled out that these metabolites influenced *S*. *litura* larval growth (Fig. 2A, see Supplementary Fig. S2C–E).

Thus, UV-B enhances tobacco resistance to *S*. *litura* in a JA pathway-dependent manner by elevating the levels of TPIs and HGL-DTGs and probably other defensive metabolites.

### JA pathway is required for enhancing rice and maize defense against insects by UV-B

**Figure 3:**
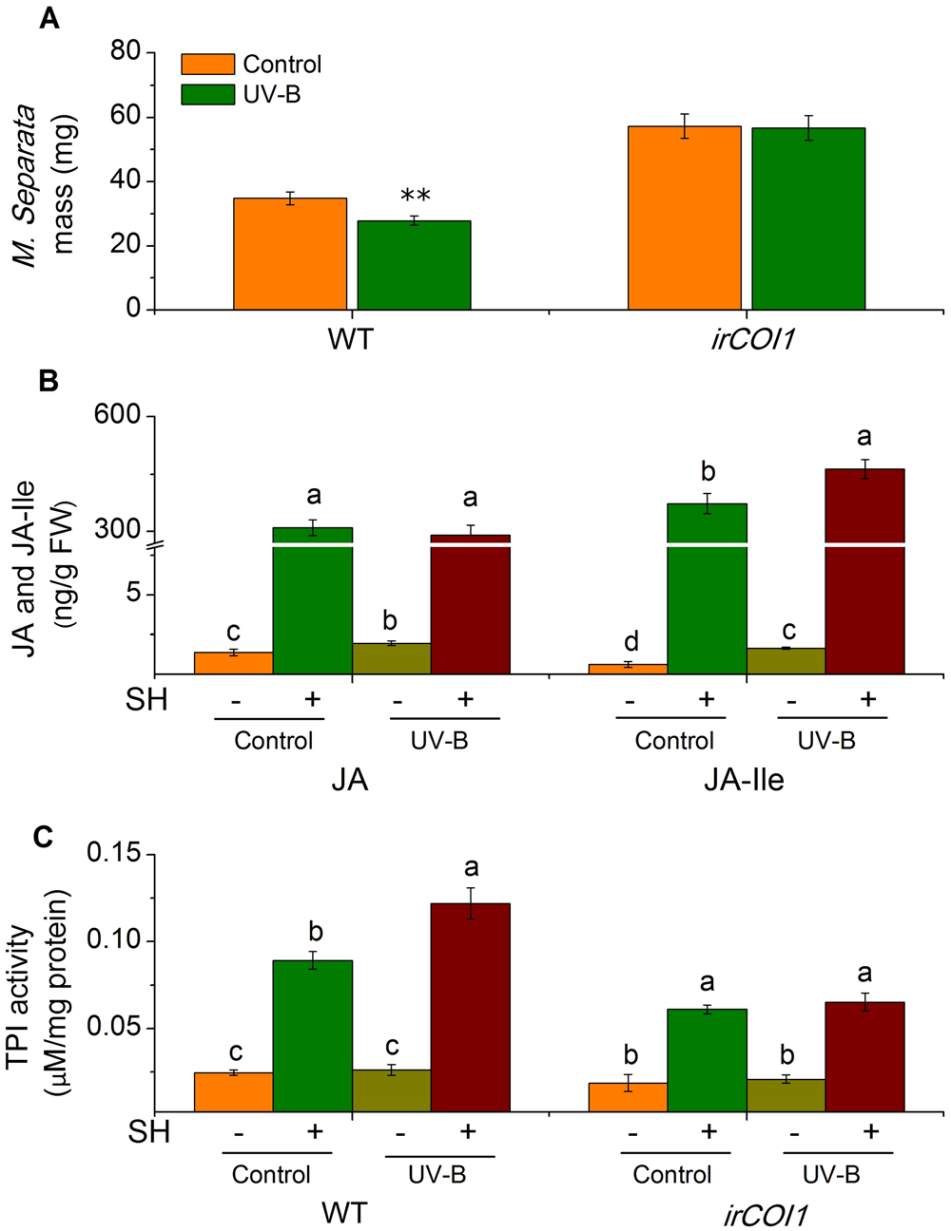
UV-B elevates rice resistance to *M*. *separata*. (**A**) Mean (±SE) *M*. *separata* masses, 8 days after feeding on control and UV-B-pretreated wild type (WT) and irCOI rice plants. Control and UV-B-pre-exposed WT and irCOI1 rice plants were treated with simulated herbivory (SH+) or left untreated (SH−), and the levels of JA and JA-Ile (**B**), and TPI activity were analyzed in samples collected at 2 h (for B) or 48 h (for C). For A, n = 30–50; for B–C, n = 6–8. Asterisks (**P < 0.01, Student’s *t*-test) and different letters (*P* < 0.05, Duncan’s multiple range test) indicate significant differences between treatments within the same genotype (for A, C) or group of compounds (for B).

We investigated whether UV-B also positively affects plant defense against insect larvae in monocots. *M*. *separata* caterpillars fed for eight days on rice plants previously treated with UV-B (3 kJ m^−2^d^−1^ for 5 d) were 20% smaller than those that fed on controls (Fig. 3A). Again, we examined the function of JA in UV-B-enhanced defense using an irCOI1 rice line, in which the JA signaling receptor *OsCOI1* was silenced by RNAi. Consistent with previous findings, *M*. *separata* gained the same weight on irCOI1 plants regardless of UV-B pretreatment (Fig. 3A). However, compared with those in the control group, rice plants in the UV-B-pretreated group did not show different levels of JA, but elevated JA-Ile (24%) contents were detected (Fig. 3B). The transcript levels of *OsLOX* (JA biosynthesis-related) and *OsJAR1* (JA-Ile biosynthesis-related) were both augmented in the UV-B-pretreated group (see Supplementary Fig. S3A).

When the defense-related secondary metabolites were analyzed, we found that although the basal TPI levels were not different, after SH treatment, 37% higher TPI activity was detected in the UV-B-pretreated rice plants, and TPI activity was similar in UV-B-pretreated and control irCOI1 rice plants (Fig. 3C). Other defense-related secondary metabolites including *p*-coumaroylputrescine, feruloylputrescine, and caffeoylputrescine, which were shown to be effective against the brown planthopper in rice and against *Manduca sexta* in *N*. *attenuata* ^26,27^, were also measured. Although their concentrations all increased in UV-B-pretreated plants, after SH, none of them showed higher levels in UV-B-pretreated plants than in the control plants (see Supplementary Fig. S3), suggesting that these metabolites are not important for UV-B-enhanced rice defense against *M*. *separata*. It should be noted that in irCOI1 plants, *p*-coumaroylputrescine, feruloylputrescine, and caffeoylputrescine accumulated almost to the same or even higher levels than in the WT plants (see Supplementary Fig. S3), indicating that the regulation of those compounds is independent of the JA signaling pathway. Moreover, *Chilo suppressalis* (rice striped stem borer, SSB) infestation induced higher levels of JA-Ile but not JA in the UV-B-pretreated rice plants and in turn elicited higher TPI activity (see Supplementary Fig. S4A–C) and SSB gained less weight on the UV-B-pretreated plants (see Supplementary Fig. S4D), supporting the findings that JA-Ile-regulated TPI activity plays an important role in rice defense against SSB^28^. Thus, in rice the UV-B-enhanced herbivore resistance is controlled by the JA pathway as well, probably mainly by enhancing TPI activity.

**Figure 4:**
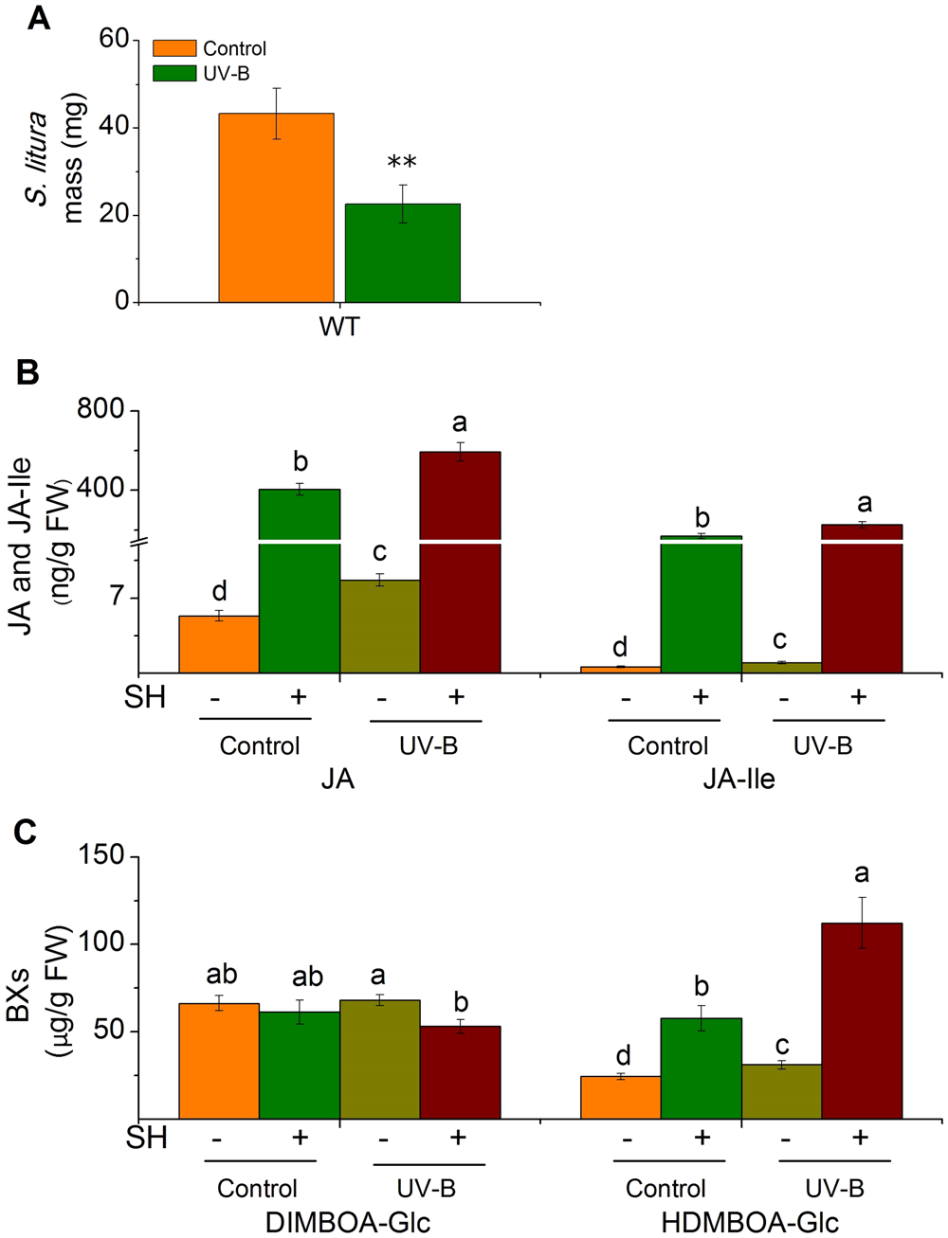
UV-B elevates maize resistance to *S*. *litura*. (**A**) Mean masses (±SE) of *S*. *litura* insects, 8 days after feeding on control and UV-B-pretreated maize plants. Control and UV-B-pre-exposed maize plants were treated with simulated herbivory (SH+) or left untreated (SH−), and the levels of JA and JA-Ile (**B**) and accumulation of BXs were analyzed in samples harvested at 2 h (for B) or 48 h (for C). For A, n = 30–50, for B–C, n = 6–8. Asterisks (**P < 0.01, Student’s *t*-test) represent significant differences between treatments, and different letters (*P* < 0.05, Duncan’s multiple range test) indicate significant differences between treatments within the same group of compounds.

Similarly, *S*. *litura* larvae gained much less weight (48%) on maize plants that were pretreated by UV-B, compared with those grew on control plants (Fig. 4A). The levels of JA and JA-Ile were all enhanced in maize plants pretreated with UV-B (Fig. 4B), and so were the transcripts levels of *ZmLOX8* and *ZmJAR1*, important genes involved in JA and JA-Ile biosynthesis in maize (see Supplementary Fig. S5A,B). Levels of two major defensive benzoxazinoids (BXs), 2,4-dihydroxy-7-methoxy-1,4-benzoxazin-3-one glucoside (DIMBOA-Glc) and 2-hydroxy-4,7-dimethoxy-1,4-benzoxazin-3-one glucoside (HDMBOA-Glc), which are anti-*S*. *littoralis* metabolites^29^, were determined: the contents of DIMBOA-Glc were not influenced by UV-B pretreatment or SH (Fig. 4C), but the HDMBOA-Glc levels were augmented by SH (32%), UV-B (64%), and UV-B pretreatment followed by SH (191%) (Fig. 4C), indicating that UV-B also elevates maize resistance to *S*. *litura*. Compared with the control group, in the UV-B-pretreated maize plants, simulated *M*. *separata* herbivory induced higher JA and JA-Ile (38 and 25%) and 39% more HDMBOA-Glc, regardless levels of DIMBOA-Glc were not affected by UV-B pretreatment or SH (see Supplementary Fig. S6A–D); however, *M*. *separata* gained equal weihgt on these two groups of maize (see Supplementary Fig. S6E). It is possible that *M*. *separata* is not sensitive to HDMBOA-Glc.

## Discussion

As a part of natural sunlight, UV-B inevitability irradiates green plants in nature. Previous studies have shown that UV-B increases plant defense against pathogens and insects, and this is correlated with UV-B-induced accumulation of defense-related metabolites (sinapate, HGL-DTGs, and flavonoids, for instance) in the epidermal tissues^13,15,16,24^. Here, using several dicotyledonous and monocotyledonous plant species and genetically modified plants, we demonstrate that UV-B increases plant resistance to lepidopteran larvae by enhancing the levels of JA and/or JA-Ile, which in turn increases defense-related secondary metabolites, and this mechanism seems to be well conserved.

In response to UV-B, plants may activate two pathways^3,11^: one is the photomorphogenic pathway, which functions through UVR8 with the collaboration of the elongated hypocotyl5 (HY5) transcription factor and constitutive photomorphogenesis1 (COP1), and the other is the nonspecific pathway, which is involved in repair of DNA damage and ROS (reactive oxygen species), MAPK, JA, and ethylene signaling^11,23,30,31^. We found that Arabidopsis UVR8 is not required for UV-B-enhanced resistance to *S*. *litura*. Thus, the UV-B-regulated defense against insects is probably controlled by the nonspecific signaling pathways, and among these, the JA pathway plays a critical role. Measurements of JA/JA-Ile and analyses on mutants or RNAi plants impaired in JA biosynthesis or perception indicated the critical role of the JA pathway in UV-B enhancing of plant defenses against insects, although how UV-B affects the JA pathway remains elusive.

Compared with those on WT plants, *S*. *litura* feeding on gs4 mutant gained twice as much weight (Fig. 1A), thus GS contents are important for the resistance of Arabidopsis plants to *S*. *litura*; however, the gs4 mutant nevertheless exhibited UV-B enhancing of resistance to *S*. *litura* (Fig. 1A). Results from the *dde-2* mutant demonstrate that the JA pathway is required for the UV-B-regulated response (Fig. 1A,D). Based on these analyses, it is likely that UV-B enhances Arabidopsis defense against *S*. *litura* by increasing herbivory-induced GS contents and an unknown JA-regulated defensive metabolite(s). We found a strong increase of phenolics (chlorogenic acid) and phenolamides (feruloylputrescine), in tobacco and rice plants respectively, which likely function as sunscreen to absorb UV-B^11^. However, phenolics and phenolamides in tobacco and rice seem not to be regulated by the JA pathway, because irAOC tobacco and irCOI1 rice plants showed similar levels of chlorogenic acid and feruloylputrescine as those in the WT plants (see Supplementary Figs S2C and S3B). It is possible that these compounds contribute to UV-B-enhanced resistance in plants defense against other insects. This was supported by our finding that UV-B-treated maize did not have elevated resistance to *M*. *separata*, but exhibited elevated defense against *S*. *litura*.

*Nicotiana attenuata* plants treated with narrowband UV-B (~ 313 nm) did not show augmented levels of JA and JA-Ile after SH under glasshouse conditions^32^; in contrast, ambient UV-B, SH-treated field-grown *N*. *attenuata* had higher JA and JA-Ile levels compared to those which did not receive UV-B^15^. Our data also showed that cultivated tobacco had enhanced resistance to *S*. *litura* after being treated with broadband UV-B. These differences may have resulted from the different UV-B light sources, i.e. the energy density of the UV-B light from the narrowband UV-B source might be quite high and therefore could cause damages to plants and compromise the synergistic effect of UV-B for defense against insects. To minimize the risk of possible cellular damages resulted from continuous UV-B radiation, in this study, the daily dosages of UV-B were not continuously applied to the plants, but were applied during three short time periods. This relatively mild manner of UV-B treatment might also have partly accounted for the increased SH-induced JA levels in our UV-B-treated Arabidopsis, tobacco, rice, and maize, compared with their respective control plants. Whether plants respond to insect feeding with different levels of JA, after being treated with continuous and periodic UV-B irradiation, respectively, should be examined. Moreover, how solar UV-B or artificial broadband UV-B elevates plant defense against insects, especially how UV-B affects plant JA biosynthesis, is interesting to explore.

The function of UV-B in influencing different plant species against piercing-sucking herbivores (aphids, thrips, and mirid bugs) has been demonstrated^15,16,32^. Here, we reveal the importance of JA pathway in UV-B enhancing plant resistance to lepidopteran larvae, and importantly, our data from Arabidopsis, tobacco, rice, and maize suggest that the function of UV-B in elevating plant defense against insects is likely well conserved in both monocots and dicots. Plants are exposed to UV-B from the moment they emerge from soil. The long-term effect of UV-B radiation on plant fitness should be further studied in natural conditions: under minimal environmental stresses, such as artificial climate chambers or glasshouses, plants exposed to UV-B (even mild dosages) may have decreased fitness, due to resource allocation to the production of sunscreen metabolites; however, it is conceivable that in nature under attack from insects and pathogens, long-term UV-B exposure increases plant defense levels, since the benefit brings about by UV-B likely overcomes the disadvantage of resource allocation to biosynthesis of defensive metabolites. Glasshouse agriculture has become an essential part of agriculture in almost all countries. However, the ceiling and wall materials, which are mainly glass and plastic films, may partly or completely block the UV spectrum of the sunlight. Thus, for greenhouse agriculture, supplementation of UV-B broad-spectrum lights may increase plant resistance to a wide range of insects, decreasing the usage of chemical pesticides. This point should be further examined in real greenhouse agricultural settings.

## Materials and Methods

### Plant materials and growth conditions

*Arabidopsis thaliana* (Col-0 background, including all mutants) seeds were vernalized for three days in darkness at 4 °C and germinated on petri dishes (1/2 Murashige Skoog [MS] medium). Seven days later, seedlings were transferred to individual pots and kept in the chamber at 22 °C under a 16-h-light/8-h-dark cycle with 150 µmol/m^2^/s photosynthetically active radiation (PAR; measured with LI-1400 DataLogger) supplied by eight lamps (Philips Essential TL5 28 W/865). Four weeks old rosette-stage plants were used in all experiments. UV-B receptor mutant *uvr8-2* (SALK_033468)^33^ was obtained from NASC (http://arabidopsis.info); *dde2-2* mutant was obtained from ABRC (http://arabidopsis.org); *gs4* mutant were kindly provided by Prof. Philippe Reymond^25^.

The partial tobacco (cv. Samsun) *NtAOC* sequence was amplified by PCR and cloned into the pB7GWIWG2(II) vector, and irAOC plants were created by *Agrobacterium*-mediated transformation as described previously^34^. The silencing efficiently (measured with wounding induced JA and JA-Ile levels) of irAOC tobacco plants can be found in Supplementary Fig. S7. The gene accession number and primers used can be found in Table S1. Rice (cv. Nipponbare) and irCOI1 line were offered by Prof. Yinong Yang^35^, and were cultivated hydroponically. The maize inbred line A188 was used in this study. Tobacco, rice, and maize were grown in a glasshouse, in which UV light was filtered out by the wall and ceiling materials (polycarbonate twin-wall hollow sun sheets), under controlled conditions with 1100-1500 µmol m^−2^ s^−1^ PAR at plant growing level (20–28 °C, day length 16 h; we used natural light, but light from sodium lamps [Philips Sun-T Agro] was supplied, in case dark days and short-day length). For tobacco and rice, six-week-old plant were used and V4-staged (around 4 weeks) maize plants were used.

### Plant treatments

Eggs of *S*. *litura*, *Mythimna separata*, *Chilo suppressalis* were purchased from Beijing Genralpest Company (http://www.genralpest.com) and newly hatched larvae were used for all experiments. *S*. *litura* neonates were not directly introduced to tobacco and maize due to high mortality rates. Instead, neonates were reared on an artificial diet for five days prior to transfer to these plants.

UV-B treatments were performed using UV fluorescent lamps (Philips TL 40 W/12, broadband) as described by Ulm *et al*.^36^. Based on the fact that 1.1 kJ m^−2^ d^−1^ UV-B treatment induced increased expression levels of Arabidopsis *PDF1*.*2* ^37^ and 0.9 kJ m^−2^ d^−1^ UV-B treatment induced GS accumulations in broccoli sprouts^38^, in this study, 1 kJ m^−2^ d^−1^ biologically effective daily UV-B dose (BE-UV-B, calculated using the plant action spectrum normalized at 300 nm)^39^, was applied to Arabidopsis which measured by UV light meter (UV-297, Lutron Electronic Enterprise). For tobacco, rice, and maize, 3 kJ m^−2^d^−1^ BE-UV-B were applied which were comparable to the UV-B dosage previously used for broccoli and *N*. *attenuata* treatment^18,32^. The BE-UV-B irradiance integrated between 290 and 315 nm was 318 mW m^−2^. In order to reduce the risk of cellular damages caused by continuous UV radiation, the running time of the UV-B fluorescent lamps was automatically controlled by an electronic timer (AL-06, Yuyao Electrical Appliance Co., Ltd.), allowing the UV-B fluorescent lamps to be turned on three times each day starting at 10 am, 1 pm, and 4 pm; for Arabidopsis plants, the UV-B lamps were turned on for 17.4 min each time (52.4 min in total each day to reach the dosage of 1 kJ m^−2^), and tobacco, rice, and maize were irradiated for 52.4 min each time (157.2 min in total each day to reach the dosage of 3 kJ m^−2^). The UV fluorescent lamps were turned off between these treatment times. Plants were irradiated five days before herbivory or treatments and showed no visible damages caused by UV-B treatment during all experiments processes. Both control and UV-B treated groups received the same PAR, even during UV-B treatment, and plants of different groups were isolated with polycarbonate twin-wall hollow sun sheets (Shanghai Pincheng Plastics Co., China), which completely filter out UV-B, to ensure no accidental UV-B radiation to the control group.

Simulated herbivory treatment was performed at 12 pm by rolling a fabric pattern wheel along the midvein two times each side and immediately rubbing 20 μl oral secretion (collected from 3rd instar caterpillars feeding on the respective plants) of the respective insects to the wounds. For determination of insect growth (caterpillar performance), control or UV-B treated plants were infested with 30–50 caterpillars and caterpillar weight was measured on the designated days.

### Quantification of phytohormones and secondary metabolites

JA and JA-Ile were analyzed as described previously^40^. Briefly, one milliliter of ethyl acetate spiked with 100 ng D_6_-JA and D_6_-JA-Ile were added to approximately 100 mg of ground leaf samples. After 10 min of vortexing, samples were centrifuged at 15,000 g for 10 min, the supernatants were transferred to fresh tubes and evaporated to dryness on a vacuum concentrator (Eppendorf). The residues were resuspended with 0.5 mL of 70% methanol (v/v) and analyzed on an UPLC-MS/MS (LCMS-8040 system, Shimadzu). Quantification of metabolites in Arabidopsis, tobacco, rice, and maize were performed on an UPLC-MS/MS (LCMS-8040 system, Shimadzu) following methods described previously^7,15,29,41^. Standards used for Arabidopsis, tobacco, rice, and maize were kindly provided by Dr. Michael Reichelt, Profs. Ian T. Baldwin, Ivan Galis, and Matthias Erb, respectively. A radial diffusion assay described by van Dam *et al*.^42^ was used to determine the activity of trypsin proteinase inhibitors.

### qRT-PCR analysis

Total RNA isolation and cDNA synthesis were performed as described previously^43^. *PP2AA3* (for Arabidopsis), *actin* (for rice and maize), and *elongation factor* (for tobacco) were used for normalizing cDNA concentrations. Primer sequences used for qRT-PCR are listed in Table S1.

### Statistical analysis

All statistics were carried out with the Statistical Product and Service Solutions (SPSS) package. Differences in all data were determined by Student’s *t*-test or one-way ANOVA. If the ANOVA was significant (*P* < 0.05), Duncan’s multiple range test was used to detect significant differences between groups.

## Electronic supplementary material

Supplementary Information
