# Supplementary material for: Ultraviolet-B enhances the resistance of multiple plant species to lepidopteran insect herbivory through the jasmonic acid pathway
Source: Sci Rep. 2018 Jan 10;8:277. doi: 10.1038/s41598-017-18600-7 (PMC5762720; doi:10.1038/s41598-017-18600-7)
Supplement: Supplementary file 1 — Supplementary Information [file 41598_2017_18600_MOESM1_ESM.pdf]

Supplementary Information

# Ultraviolet-B enhances the resistance of multiple plant species to lepidopteran insect herbivory through the jasmonic acid pathway

Jinfeng Qi[a], Mou Zhang[b], Chengkai Lu[a], Christian Hettenhausen[a], Qing Tan[a], Guoyan Cao[a], Xudong Zhu[c], Guoxing Wu[b], and Jianqiang Wu[a, *]

[a] Department of Economic Plants and Biotechnology, Yunnan Key Laboratory for Wild Plant Resources, Kunming Institute of Botany, Chinese Academy of Sciences, Kunming 650201, China

[b] College of Plant Protection, Yunnan Agriculture University, Kunming 650201, China

[c] State Key Laboratory of Rice Biology, China National Rice Research Institute, Hangzhou 31006, China

* Corresponding author: Jianqiang Wu

Phone/Fax: +86-871-65229562

Email: wujianqiang@mail.kib.ac.cn

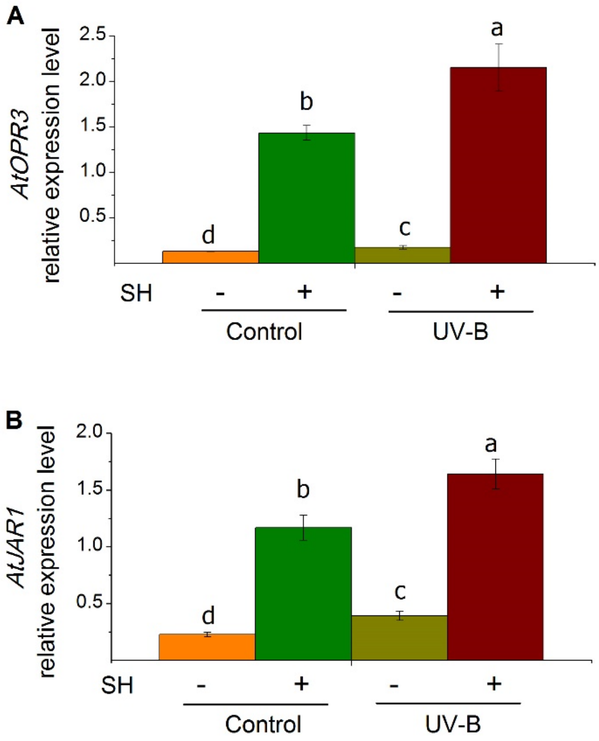


**Supplementary Figure 1.** UV-B treatment elevates the expression levels of *AtOPR3* and *AtJAR1*.

Control and UV-B-pre-exposed Arabidopsis plants were treated with simulated herbivory (SH +) or left untreated (SH -), and the levels (mean ± SE) of *AtOPR3* (A) and *AtJAR1* (B) were determined in samples collected at 2 h. n = 6-8. Different letters ($P$ < 0.05, Duncan's multiple range test) indicate significant differences between groups.

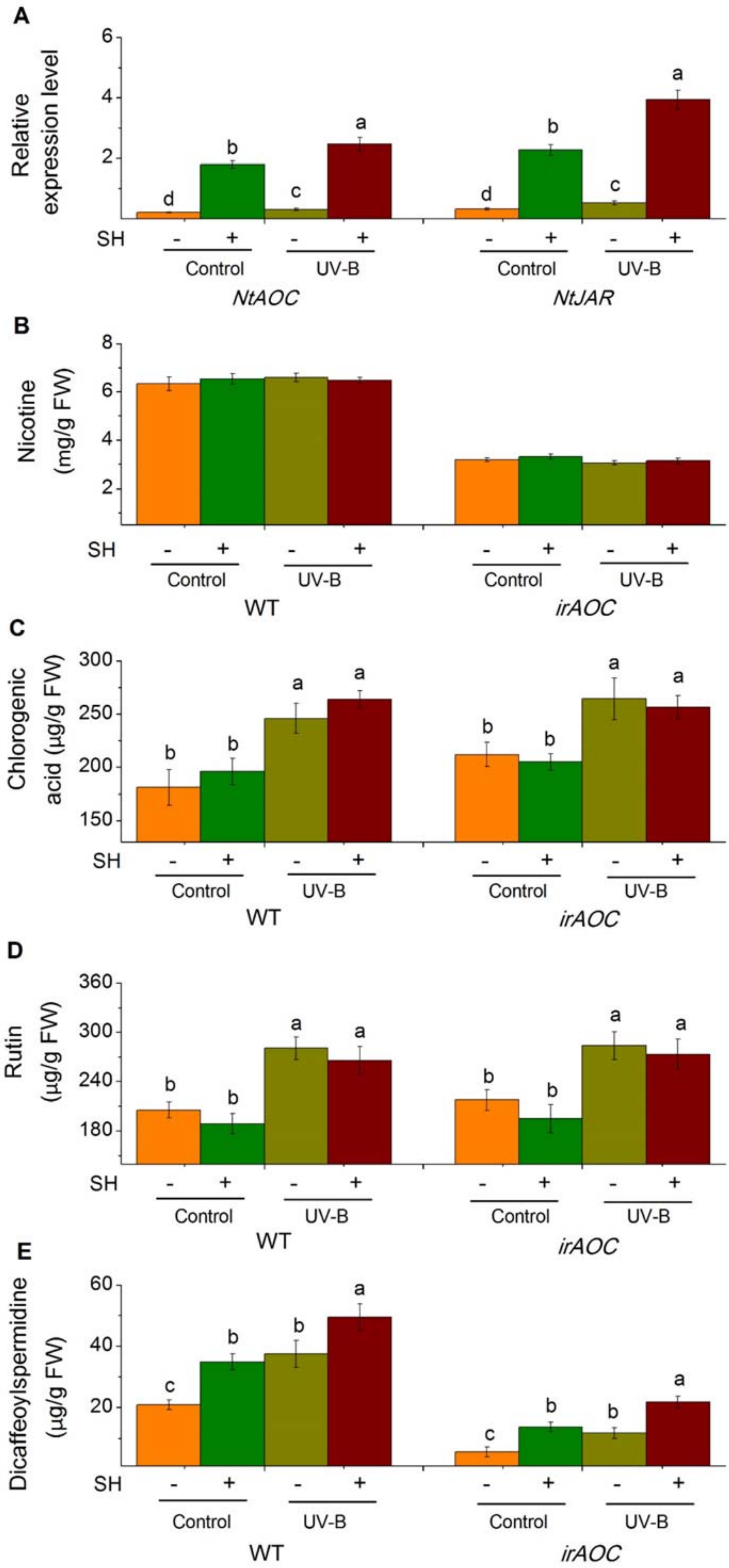
A
Relative expression level
6
4
2
d
b
c
a
d
b
c
a
SH
- + - +
Control
UV-B
NtAOC
- + - +
Control
UV-B
NtJAR
B
Nicotine (mg/g FW)
8
6
4
2
SH
- + - +
Control
UV-B
WT
- + - +
Control
UV-B
irAOC
C
Chlorogenic acid (μg/g FW)
300
250
200
150
b
b
a
a
b
b
a
a
SH
- + - +
Control
UV-B
WT
- + - +
Control
UV-B
irAOC
D
Rutin (μg/g FW)
360
300
240
180
b
b
a
a
b
b
a
a
SH
- + - +
Control
UV-B
WT
- + - +
Control
UV-B
irAOC
E
Dicaffeoylspermidine (μg/g FW)
60
40
20
c
b
b
a
c
b
b
a
SH
- + - +
Control
UV-B
WT
- + - +
Control
UV-B
irAOC

**Supplementary Figure 2.** Gene expression and secondary metabolites of *N. tabacum* in response to UV-B treatment and simulated herbivory.

Control and UV-B-pre-exposed wild-type (WT) and irAOC tobacco plants were treated with simulated herbivory (SH +) or left untreated (SH -), and levels (mean ± SE) of *NtAOC1* and *NtJAR4* (A) and the contents of secondary metabolites nicotine (B), chlorogenic acid (C), rutin (D), and dicaffeoyl spermindine (E) were determined in samples collected at 2 h (for a) or 48 h (for b-e). n = 6-8. Different letters ($P < 0.05$, Duncan’s multiple range test) indicate significant differences between treatment groups within the same gene (for A) or genotype (for B-E).

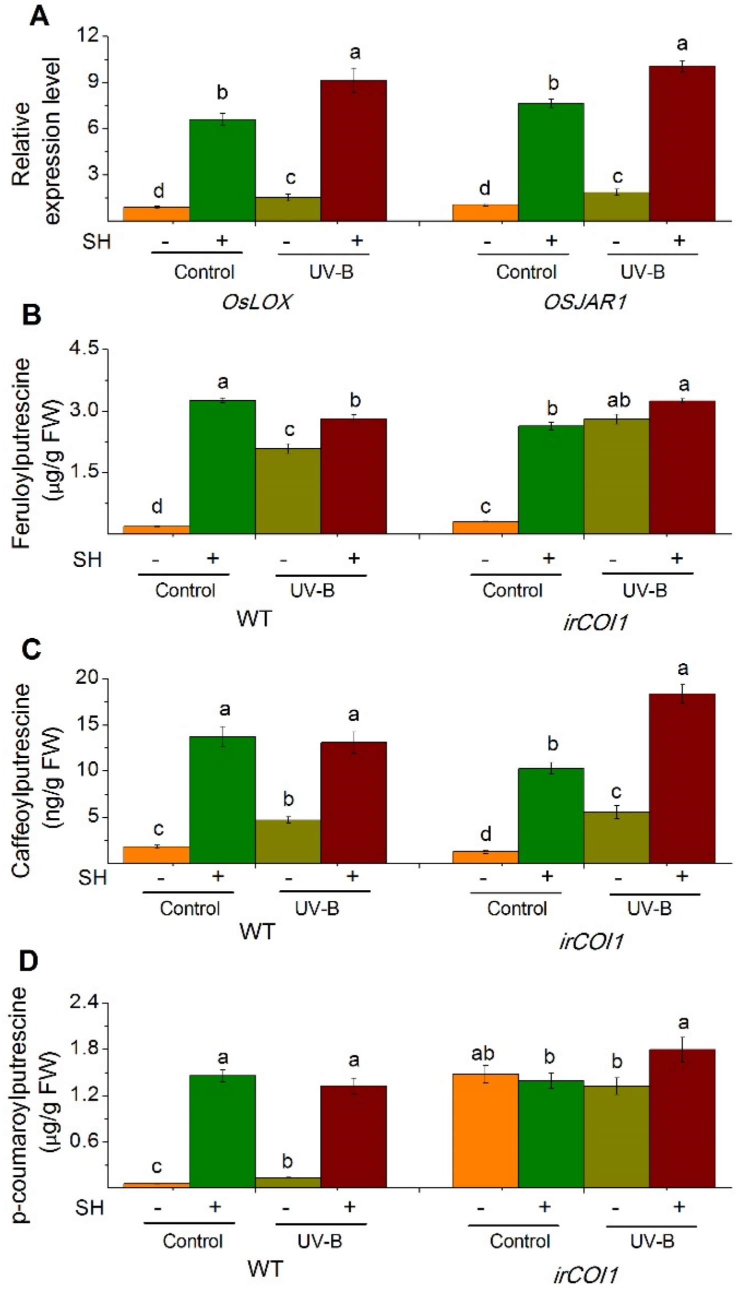


**Supplementary Figure 3.** Gene expression and secondary metabolites of rice in response to UV-B treatments and simulated herbivory.

Control and UV-B-pre-exposed wild-type (WT) and irCOI1 rice plants were treated with simulated herbivory (SH +) or left untreated (SH -), and the levels (mean ± SE) of *OsLOX*, and *OsJAR1* (A) and the contents of secondary metabolites feruloylputrescine (B), caffeoylputrescine (C), and *p*-coumaroylputrescine (D) were

determined in samples collected at 2 h (for A) or 48 h (for B-D). n = 6-8. Different letters (*P* < 0.05, Duncan’s multiple range test) indicate significant differences between treatment groups within the same gene (for A) or genotype (for B-D).

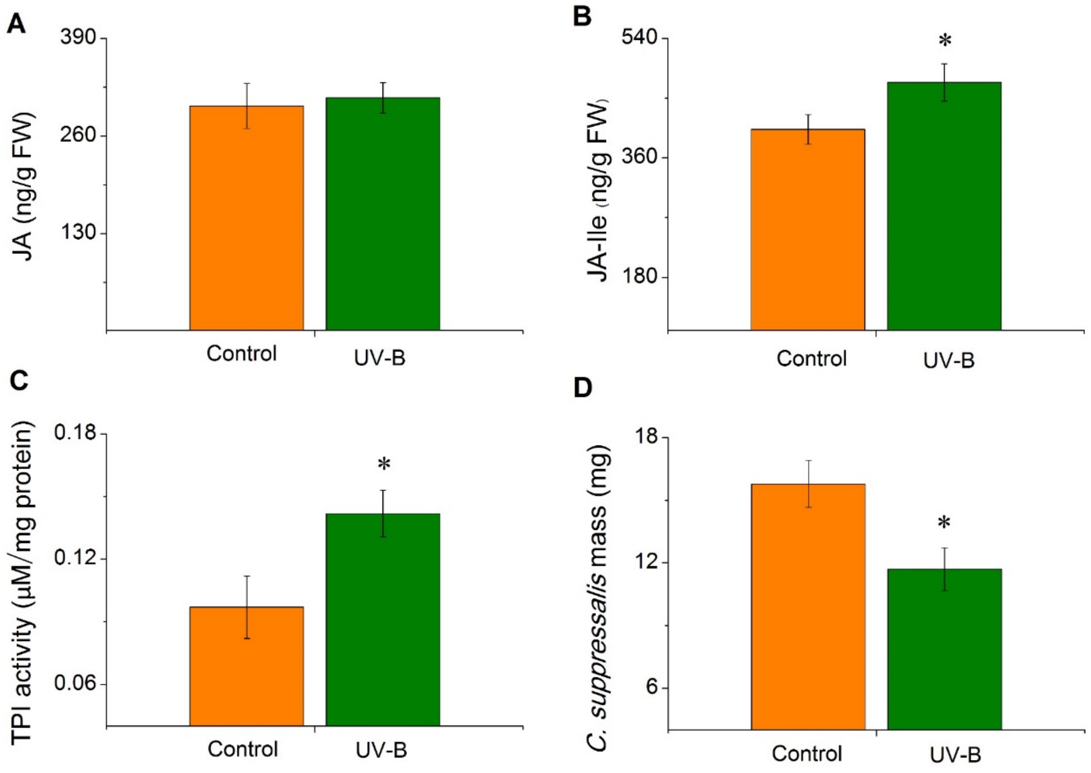


**Supplementary Figure 4.** UV-B-enhanced rice defense traits against the striped stem borer (SSB) *C. suppressalis*.

Control and UV-B-pre-exposed rice plants were infested by SSB (A-C) and levels (mean ± SE) of JA (A), JA-Ile (B), and TPI activity (C) were determined in samples collected at 2 h (for A and B) or 48 h (C); mean masses (± SE) of SSB after 12 days feeding on control and UV-B-pretreated WT rice plants (D). For A-C, $n$ = 6-8, for D, $n$ = 30-50. Asterisks (* $P < 0.05$, Student's $t$ test) indicate significant differences between treatments.

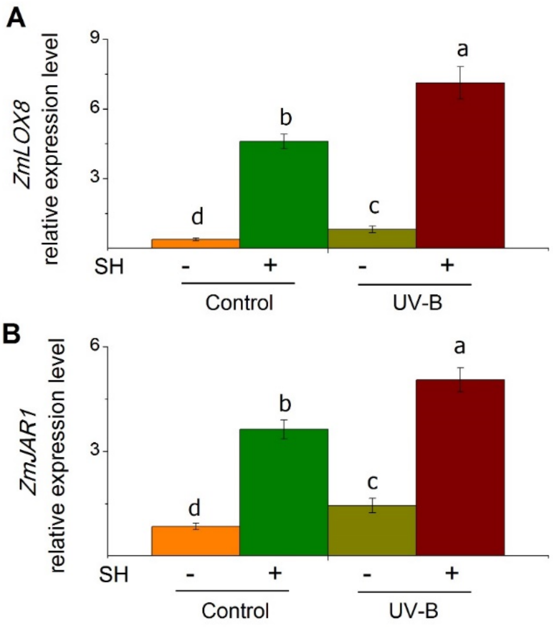


**Supplementary Figure 5.** Responses of maize to UV-B pre-treatment and simulated *M. separata* feeding.

Control and UV-B-pre-exposed maize plants were treated with simulated herbivory (SH +) or left untreated (SH -), and the levels (mean ± SE) of *ZmLOX8* (A) and *ZmJAR1* (B) were determined in samples collected at 2 h. $n$ = 6-8, letters ($P < 0.05$, Duncan's multiple range test) indicate significant differences between treatments.

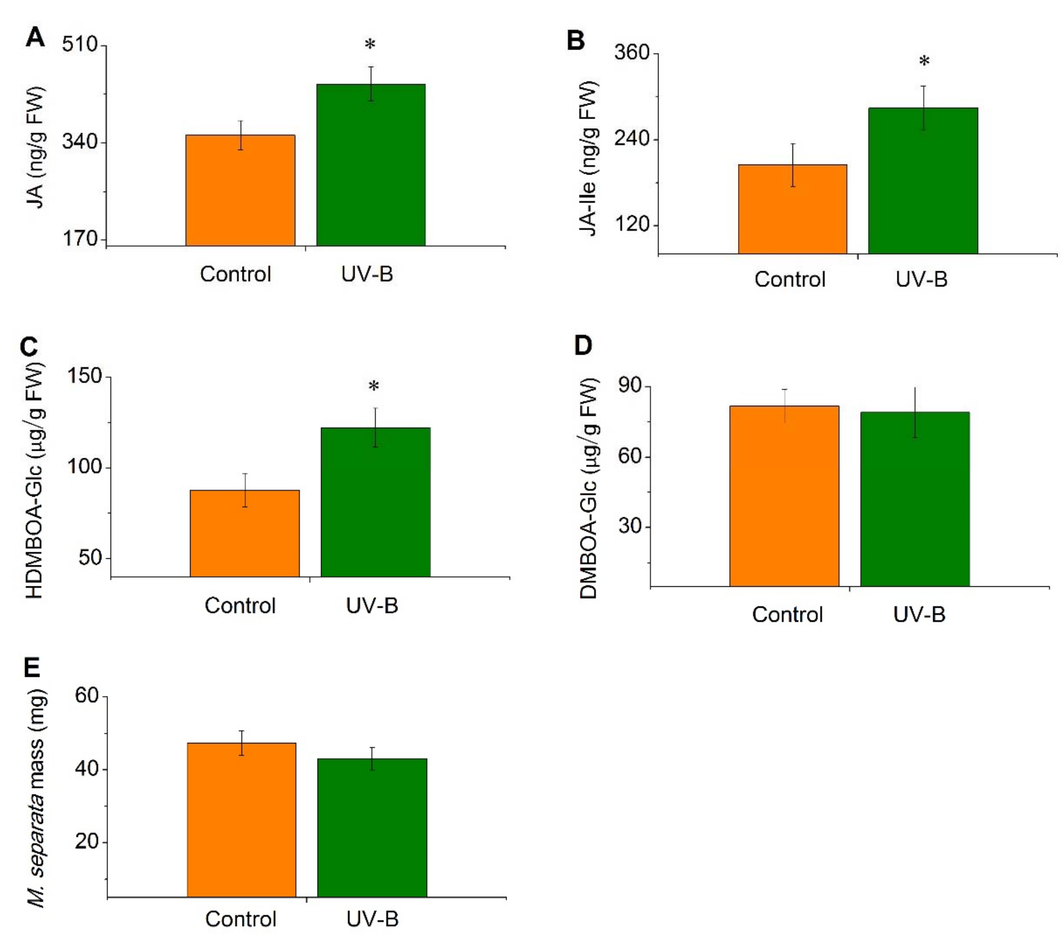


**Supplementary Figure 6.** Growth of *M. separata* on control and UV-B--pre-treated maize plants.

Both control and UV-B-pre-exposed (UV-B) maize plants were treated with simulated herbivory, and contents (mean ± SE) of JA (A), JA-Ile (B), HDMBOA-Glc (C) and DMBOA-Glc (D) were determined in samples collected at 2 h (for A and B) or 48 h (C); mean masses (± SE) of *M. separata* 8 days after feeding on control and UV-B-pretreated maize (E). For a-c, $n$ = 6-8, for D, $n$ = 30-50. Asterisks (* $P < 0.05$, Student's $t$ test) indicate significant differences between treatments.

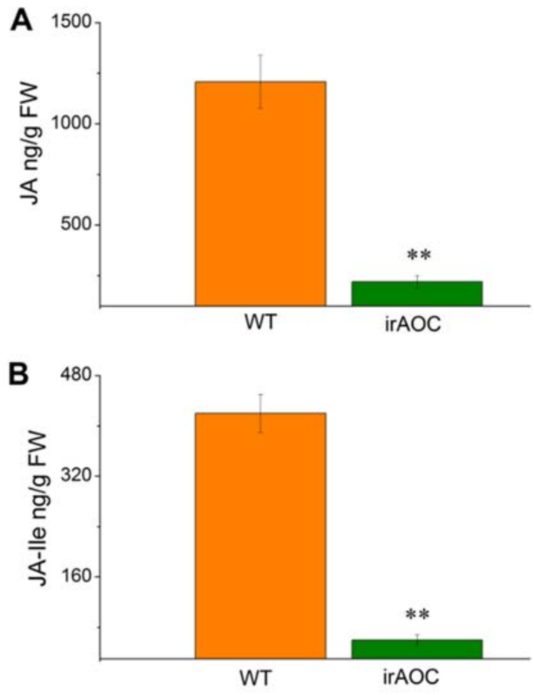


**Supplementary Figure 7.** Silencing efficiently of irAOC tobacco plants.

Wild-type (WT) and irAOC plants were treated with wounding, and the levels (mean ± SE) of JA (A), JA-Ile (B) were determined in samples collected at 1 h. Asterisks (** $P < 0.01$, Student's *t*-test, n = 5) indicate significant differences between genotypes.

**Supplementary Table 1.** Primers used in this study

| Gene name | Gene ID | Forward Primer (5' to 3') | Reverse Primer (5' to 3') |
|---|---|---|---|
| *AtPP2AA3* | AT1G13320 | ACCTGCGGTAATAACTGCATCTAA | ACCAAGCATGGCCGTATCAT |
| *AtOPR3* | AT2G06050 | GAAGATTCGATCTCTCTCATCG | GGTCCGTTGAGCATAATACTC |
| *AtJAR1* | AT2G46370 | AGACGATCCGCACCAAATGT | TTTGGCACATACGGCTCCAT |
| *NtACT* | XM_009633597 | TGATAACGGAACAGGAATGG | TCGAGGTCGACCAACAATAC |
| *NtJAR* | DQ359729.1 | GCCTCCCGAGCTTGTTACAT | GACCGTCTAAATTTTCCATGAGA |
| *NtAOC* | NM_001324978.1 | GAGCCAGCACCTGAAGCTAA | TTCTCCGGAAATGACCCCAC |
| *NtAOC* for RNAi | NM_001324978.1 | CACCTCCACCAACTCCAAGT | TGCTGCAATTTCACTTGACC |
| *OsACT* | Os03g50885 | TGGACAGGTTATCACCATTGGT | CCGCAGCTTCCATTCCTATG |
| *OsLOX* | Os08g39840 | TCAAGGAGATCGAGGGCGT | TCGGAGAAGGGCTTCATCAG |
| *OsJAR1* | Os05g50890 | AAGGTTTGTGAACCCATCAAACAGC | AATAATACTTTGCAGCACTTGTTACG |
| *ZmACT* | GRMZM2G006765 | GGAGCTCGAGAATGCCAAGAGCAG | GACCTCAGGGCATCTGAACCTCTC |
| *ZmJAR1* | GRMZM2G091276 | GGTCCATGGAGCCATACCTG | TAGAGCCGACCCATCCTTCA |
| *ZmLOX8* | GRMZM2G104843 | CATCAAGAGTAAGCCAGCGC | GTTCGATGTTGACGGGGTTG |
